# Editorial: Double-edged swords: Important factors connecting metabolic disorders and cancer development – from basic research to translational applications

**DOI:** 10.3389/fendo.2023.1168700

**Published:** 2023-03-02

**Authors:** Che-Pei Kung, Thibaut Barnoud, Cong-Hui Yao, Maureen E. Murphy

**Affiliations:** ^1^ Division of Molecular Oncology, Department of Medicine, School of Medicine, Washington University in St. Louis, Saint Louis, MO, United States; ^2^ Department of Biochemistry and Molecular Biology, College of Medicine, Medical University of South Carolina, Charleston, SC, United States; ^3^ Department of Cell Biology, Blavatnik Institute, Harvard Medical School, Boston, MA, United States; ^4^ Program in Molecular and Cellular Oncogenesis, The Wistar Institute, Philadelphia, PA, United States

**Keywords:** metabolism, cancer, translational research, MetS (metabolic syndrome), microbiome, obesity, p53, metabolomics

The relationship between metabolic dysfunction and cancer development has been informed by research of epidemiology, endocrinology, cancer biology, chemistry, genomics, and metabolomics. By revealing both distinctive and shared mechanisms, the articles in this issue, including both original research studies and reviews, point to opportunities to discover new diagnostic and therapeutic strategies.

The relationship between metabolic syndromes (MetS) and cancer is multi-faceted (1). One field of interest is to link existing MetS with cancer prognosis. In *“Effects of Metabolic Syndrome and Its Components on the Prognosis of Endometrial Cancer”*, Yang et al. explored the effects of MetS on the prognosis of endometrial cancer (EC) in over 500 patients, and found that combining high-density lipoprotein cholesterol with tumor stage and grade improved the prediction power for patient survival.

MetS also impacts other clinical outcomes in cancer patients. In *“Effect and Management of Excess Weight in the Context of Fertility-Sparing Treatments in Patients with Atypical Endometrial Hyperplasia and Endometrial Cancer: Eight-Year Experience of 227 Cases”*, Shan et al. investigated the clinical outcomes of cancer therapy and fertility-sparing treatments (FSTs) in overweight EC patients. They showed that the combination of gonadotropin-releasing hormone agonist and levonorgestrel intrauterine device resulted in better disease-free survival for overweight patients. Moreover, patients with normal weight achieved a better pregnancy rate through FSTs, while overweight patients benefited from ovulation induction to improve fertility success.

In *“Hepatocellular Carcinoma and Obesity, Type 2 Diabetes Mellitus, Cardiovascular Disease: Causing Factors, Molecular Links, and Treatment Options”*, Zhang et al. summarized the current literature linking MetS, including diabetes, obesity, and cardiovascular disease, to the development of non-alcoholic fatty liver disease and HCC. They discussed signaling pathways involved and their potential as therapeutic targets. Can we take advantage of this knowledge to develop better diagnostic tools? That is the question Cao et al. explored in “Metabolic Profiling Identified a Novel Biomarker Panel for Metabolic Syndrome-Positive Hepatocellular Cancer”, in which they used targeted metabolomic analysis to identify biomarkers for MetS-positive HCC. A panel including L-gluamic acid, pipecolic acid and alpha-fetoprotein was identified as a potential diagnostic tool for MetS-positive HCC. With recent advances in metabolomic technologies, more examples like this could become reality in aiding early diagnosis and personalized therapy of cancer (2).

Outside of clinical settings, we have also highlighted preclinical investigations studying roles of metabolic pathways in cancer progression and therapy response. The tumor suppressor p53 plays versatile roles in regulating metabolic dysfunction and cancer (3). In *“Mutant p53-microRNA-200c-ZEB2-Axis-Induced CPT1C Elevation Contributes to Metabolic Reprogramming and Tumor Progression in Basal-Like Breast Cancers”*, Wang et al. provided new insight about how mutant p53 (Mutp53) contributes to tumor progression in basal-like breast cancers. They demonstrated that Mutp53 enhances fatty acid oxidation through dysregulating the miR-200c-ZEB2 axis to induce CPT1C, resulting in epithelial-mesenchymal transition phenotypes and increased cancer stemness. These data suggest that Mutp53-driven metabolic reprogramming could be an effective therapeutic target. In a complementary Mini-Review article *“p53-Mediated Indirect Regulation on Cellular Metabolism: From the Mechanism of Pathogenesis to the Development of Cancer Therapeutics”*, Wang and Chao summarized the indirect regulation of cellular metabolism by wild-type and mutant p53 through 1) transcriptional targets, 2) protein-protein interaction with other transcription factors, and 3) other signaling pathways. These findings support the premise that improving understanding of these mechanisms can inform novel therapeutic strategies through the concept of synthetic lethality.

One of the most studied cancer-regulating metabolic mechanisms is the Warburg effect, describing cancers’ propensity to use glycolysis for their survival (4). In *“FBP1/miR-24-1/enhancer axis activation blocks renal cell carcinoma progression via Warburg effect”*, Ju et al. showed that FBP1, a rate-limiting enzyme regulating gluconeogenesis and whose downregulation in renal cell carcinoma (RCC) is linked to poor survival, can be activated to slow down RCC progression. This can be achieved through employing miR-24-1, a nuclear activating miRNA, to activate FBP1 and repress the Warburg effect in RCC cells.

Metabolic alterations also regulate treatment response of cancer. In *“Lapatinib Suppresses HER2-Overexpressed Cholangiocarcinoma and Overcomes ABCB1– Mediated Gemcitabine Chemoresistance”*, Bai et al. showed that HER2-targeting lapatinib overcomes gemcitabine resistance in cholangiocarcinoma (CCA). Gemcitabine works as a nucleoside analog to suppress cancer cell proliferation, and the authors showed that the active metabolite of gemcitabine, dFdCTP, is a substrate of ATP-binding cassette subfamily B member 1 (ABCB1). Gemcitabine-treated CCA cells develop a negative feedback loop by upregulating ABCB1 to cause gemcitabine resistance, which can be circumvented by the combination of lapatinib and gemcitabine.

The relationship between metabolism and tumorigenesis is more than one-directional. Oncogenes and tumor suppressors can also play important roles in the development of metabolic diseases (5, Chen et al.). In *“Tumor Suppressor Par-4 Regulates Complement Factor C3 and Obesity”*, Araujo et al. used both *in vitro* and *in vivo* models to show that another tumor suppressor, prostate apoptosis response-4 (Par-4), contributes to the development of obesity. Par-4 suppresses p53 and its target, complement factor c3, to regulate fat storage in adipocytes. Mice with Par-4 deletion developed obesity even with standard diet. Lower expression of Par-4 was found in obese patients and associated with increased risk of developing obesity later in life.

Finally, the microbiome can dictate our susceptibility to metabolic dysfunction and cancer (6). In *“Connecting the Dots Between the Gut–IGF-1–Prostate Axis: A Role of IGF-1 in Prostate Carcinogenesis”*, Matsushita et al. summarized the relationship between prostate cancer (PCa) and Insulin-like growth factor 1 (IGF-1) to exemplify this concept. They highlighted the recent finding that short-chain fatty acids produced by the gut microbiota increase IGF-1 production, resulting in PCa progression through activation of downstream signaling pathways (7). They suggest that specialized dietary interventions could be implemented for prevention and treatment of PCa through optimizing the gut microbiome.

With fast-growing interest from scientific communities of different disciplines to understand the relationship between metabolic disorder and cancer development, this Research Topic hopefully serves as a precursor and medium to facilitate more nuanced, even provocative discussions to re-think and innovate actionable strategies to reduce the burden of human disease.

## Author contributions

C-PK drafted the manuscript. C-PK, TB, C-HY, and MEM reviewed and revised the manuscript. All authors contributed to the article and approved the submitted version.
